# Sociodemographic and Clinical Profiles of Participants in Nova Scotia’s Rapid Access Stabilization Program and Community Mental Health Program: A Comparative Analysis

**DOI:** 10.3390/jcm14072412

**Published:** 2025-04-01

**Authors:** Medard K. Adu, Raquel da Luz Dias, Samuel Obeng Nkrumah, Belinda Agyapong, Ngozi Ezeanozie, Ejemai Eboreime, Gloria Obuobi-Donkor, Sanjana Sridharan, Jason Morrison, Bryanne Taylor, Monica MacKinnon, Mahmoud Awara, Lori Wozney, Vincent I. O. Agyapong

**Affiliations:** 1 Department of Psychiatry, Faculty of Medicine, Dalhousie University, Halifax, NS B3H 4R2, Canada; md269491@dal.ca (M.K.A.); rq691367@dal.ca (R.d.L.D.); sm416395@dal.ca (S.O.N.); ngozi.ezeanozie@nshealth.ca (N.E.); ejemai.eboreime@dal.ca (E.E.); gl246428@dal.ca (G.O.-D.); sn372939@dal.ca (S.S.); jason.morrison@nshealth.ca (J.M.); mahmoud.awara@nshealth.ca (M.A.); lori.wozney@dal.ca (L.W.); 2 Department of Psychiatry, Faculty of Medicine and Dentistry, University of Alberta, Edmonton, AB T6G 2R3, Canada; bagyapon@ualberta.ca; 3 Mental Health and Addictions Program, Nova Scotia Health, Halifax, NS B3S 0H6, Canada; bryanne.taylor@nshealth.ca (B.T.); monica.mackinnon@nshealth.ca (M.M.); 4 Research, Innovation and Discovery, Nova Scotia Health, Halifax, NS B3S 0H6, Canada

**Keywords:** rapid access, mental health, community, addiction, sociodemographic characteristics, program-specific interventions, early intervention

## Abstract

**Background/Objective:** To address the growing demand for mental health services, Nova Scotia Health introduced the Rapid Access Stabilization Program (RASP) through its Mental Health and Addictions Program (MHAP) in April 2023. RASP is designed to help reduce long wait times, frequent emergency department visits, and admissions to provide early intervention for individuals experiencing mental health problems. The RASP focuses on rapid access and early mental health intervention, aiming to prevent the worsening of patients’ symptoms, improve access to psychiatric care, and reduce service pressures on programs like the Community Mental Health Program (CMHP), which provide more extended, ongoing mental health support. This study compared participants’ sociodemographic and clinical profiles in the RASP and the CMHP. **Methods:** Data were collected from 1392 participants accessing mental health support either through the RASP or CMHP. A comparative analysis of sociodemographic factors (e.g., age, education, and income) and clinical characteristics (e.g., depression, anxiety, resilience, and substance use) was conducted. Chi-square tests and independent sample *t*-tests were used to evaluate the mean differences between the groups. **Results:** Significant sociodemographic and clinical differences emerged between the RASP and CMHP participants. The RASP group was older (M = 40.10 vs. 34.52 years) and more socioeconomically stable, with higher rates of employment (55.3% vs. 47.9%) and homeownership (36.5% vs. 17.7%). In contrast, the CMHP group had higher unemployment (25.7% vs. 16.5%) and lower income levels, with 47.5% earning <CAD 29,590 compared to 30.3% in the RASP group. Clinical profiles differed markedly: depression was more prevalent in the RASP (48.2% vs. 19.3%), whereas the CMHP had higher rates of psychosis (10.6% vs. 2.5%) and substance use disorder (7.8% vs. 1.9%). The RASP participants exhibited higher anxiety (GAD-7: M = 14.17 vs. 11.81) and depression symptoms (PHQ-9: M = 16.62 vs. 14.20) but lower resilience (BRS: M = 2.47 vs. 2.77). The CMHP participants had more adverse childhood experiences (ACE: M = 3.92 vs. 3.16) and lower suicidal intent (81.4% vs. 99.4% had no intention to act). **Conclusions:** The findings highlighted the unique profiles between the RASP and CMHP participants, suggesting the need for program-specific interventions. While the CMHP participants may benefit from integrated social support and trauma-informed care, the RASP participants may require cognitive behavioral therapy and resilience-building interventions. Tailoring mental health services to meet these unique needs could enhance program effectiveness and patient outcomes across both groups.

## 1. Introduction

Mental health disorders represent a significant public health burden in Canada, contributing to substantial personal, social, and economic costs. According to the Mental Health Commission of Canada 2017 report, it is estimated that one in five Canadians will experience a mental health issue in any given year, with conditions such as anxiety, depression, and substance use disorders being among the most prevalent [1]. In addition to the associated human suffering, untreated mental health problems increase the chances of chronic health conditions, unemployment, homelessness, and suicide [2]. The economic burden of mental illness in Canada, including health care costs, productivity losses, and social services, is estimated to exceed CAD 50 billion annually [3]. Despite these staggering figures, barriers such as long wait times, geographical limitations, and stigma continue to impede access to timely and effective mental health care services.

To address the critical gaps in mental health care access and efficiency in Nova Scotia, the Dalhousie University’s Department of Psychiatry at the Faculty of Medicine, in collaboration with the Nova Scotia Health (NSH) Central Zone, implemented the Rapid Access and Stabilization Program (RASP) [4]. The provincial Mental Health and Addiction Program follows a stepped care model, offering a continuum of services based on individual needs. This five-tier system ranges from Tier 1 (population health promotion) to Tier 5 (specialized mental health and addictions care), categorizing services by illness severity and care intensity. The RASP aligns with Tier 3 (formal mental health and addictions care), providing rapid access and stabilization for individuals with moderate-to-severe mental health challenges. It bridges primary care and higher-intensity interventions, ensuring timely support to prevent symptom escalation and hospitalization.

The program, launched in April 2023, was designed with active stakeholder engagement, including input from the NSH/Mental Health and Addictions Program (MHAP) and service users (MHAP Patient Advisory Council). Built on the principles of collaborative care, the RASP provides swift access to psychiatric care by using the traditional referral process within primary care but bypassing the need for patients to see a mental health clinician first. It focuses on rapid stabilization, ensures continuity of care, and empowers PHPs to manage patients’ mental health needs, reducing the reliance on specialist care. In February 2024, the RASP was further enhanced by integrating a digital mental health component into its model of care. This rapid access to care offers timely responses to the mental health needs of individuals, preventing the frequent use of high-cost services such as emergency department (ED) and inpatient treatments [5,6]. Text4Support [7] is a daily text messaging program offered to all patients attending the RASP as an add-on program. This addition ensures a continuum of care by allowing patients to continue their treatment seamlessly within primary care.

Data indicate that such early intervention efforts enhance mental health outcomes for individuals and their families, leading to improved quality of life, reduced disability, and heightened productivity among those affected [8]. Therefore, expanding such programs is crucial to bridging the gap between service demand and availability, ensuring that individuals receive the care they need without delays and building a more accessible and responsive mental health system in Nova Scotia.

Before their appointment with the psychiatrists, all patients accessing the RASP are scheduled to complete a baseline assessment questionnaire that captures both sociodemographic characteristics (age, sex of birth, gender, ethnicity, employment status, source of income, relationship status, housing status, and educational level) and clinical characteristics. The patient’s clinical information is collected on validated patient-completed scales.

Following each psychiatric assessment, a comprehensive report is promptly sent to the referring primary care provider within 24 h. It covers all essential evaluation elements and includes standardized rating scale scores, interpretation, and a holistic treatment plan. Primary care providers are encouraged to contact the RASP psychiatrist for inquiries and to re-refer patients if needed. Patients receive information about community resources, psychoeducation, and guidance on lifestyle modifications promoting mental well-being.

On the other hand, the CMHP offers more comprehensive, ongoing support for individuals with complex and persistent mental health disorders, such as schizophrenia, bipolar disorder, or major depression. The program provides the therapeutic services, case management, and community support that are essential for long-term management. Unlike the RASP’s short-term focus, the CMHP addresses the sustained needs of individuals, providing continuity of care over time.

The differences in service provision for these two mental health programs attract participants with varying sociodemographic and clinical profiles. These differences are essential not only for clinicians but also for policymakers and researchers aiming to optimize mental health services. Gender differences, for example, may influence engagement with types of services, while education and employment status are frequently linked to mental health outcomes [9]. Higher education levels and stable employment correlate with better mental health, whereas lower education and unemployment worsen psychological problems [9]. A comparison of the sociodemographic and clinical characteristics of the RASP and CMHP participants will reveal patterns that inform policy and service design and aid stakeholders in gaining insights into the distinct needs of these populations and the barriers they may face [10].

This study aims to explore the sociodemographic and clinical characteristics of the assisted population (RASP) and to compare these with the characteristics of individuals accessing services through the CMHP. Key factors such as age, gender, ethnicity, socioeconomic status, and the type and severity of mental health symptoms will be analyzed to highlight the distinct profiles of the populations served by each program. The goal is to better understand the distinct populations served by each program and identify potential gaps in service delivery. The comparison is descriptive and exploratory, with the intent of supporting tailored mental health service improvements. The findings will provide valuable insights to mental healthcare providers, decision-makers, and stakeholders about the unique features of these populations that need to inform care delivery. By identifying differences in characteristics, this research aims to enhance the accessibility and uniquely designed care interventions, ultimately contributing to more effective service models and better outcomes for people with mental health issues.

## 2. Method

### 2.1. Study Design

This study employed a cross-sectional, comparative design to examine the sociodemographic and clinical characteristics at program entry for individuals accessing services through the RASP and CMHP. This approach enables the identification of patterns in service utilization and informs future service delivery and policy development.

### 2.2. Study Setting

Mental health care in Nova Scotia is publicly funded and delivered by Nova Scotia Health through the MHAP, which serves residents across four administrative zones: Central, Eastern, Western, and Northern. The RASP specifically serves patients within the Central Zone (CZ), located in Halifax, the provincial capital. Established in April 2023 at the QEII Health Sciences Centre, the RASP operates as a Tier 3 model of care, aiming to expedite psychiatric assessments, provide treatment recommendations for primary care providers, and offer digital mental health supports to reduce wait times, decrease emergency department visits, and minimize inpatient psychiatric admissions. Referrals to the RASP are made by primary healthcare providers (PHPs), including nurse practitioners and general practitioners, via a centralized telephone intake system. The program operates within standard provincial intake hours.

Study participants drawn from the CMHP are individuals who consented to take part in the ongoing clinical trial that is seeking to evaluate the effectiveness of an add-on, supportive text messaging service (Text4Support) developed using cognitive–behavioral therapy (CBT) principles to augment mental health support for patients attending to or being discharged from psychiatric care in Nova Scotia, Canada [7]. Upon giving consent, participants were requested to fill out an online baseline survey encompassing sociodemographic details and validated scales measuring mental health outcomes. Additionally, a clinician-administered scale assessing suicidal risk was completed by a trained research team member either in person or via phone [7].

### 2.3. Data Collection

Sociodemographic and mental health assessment information included results from patient-completed validated scales and the psychiatry diagnosis obtained from the Text4Support database and the RASP’s paper-based baseline assessments from April 2023 to April 2024.

### 2.4. Outcome Measures

Outcome measures include the sociodemographic and clinical characteristics of the assisted population and the differences in mean scores on the Patient Health Questionnaire (PHQ-9) [11], Generalized Anxiety Disorder (GAD-7) scale, WHO 5 Well-Being Index, Brief Resilience Scale (BRS), Recovery Assessment Scale (RAS), Adverse Childhood Experiences (ACE) questionnaire, and Brief Substance Craving Scale (BSCS), as well as the prevalence of likely major depression disorder (MDD), and generalized anxiety disorder (GAD) between the RASP and the CMHP group.

The PHQ-9 was employed to evaluate participants’ depressive symptoms. This nine-item measure utilizes a four-point Likert scale ranging from 0 (not at all) to 3 (nearly every day) [11]. A score of 10 or higher suggests the presence of MDD. The PHQ-9 is recognized for its robust psychometric properties, including high reliability and validity, and it has demonstrated strong internal consistency [11]. The Cronbach alpha coefficient for the PHQ-9 is 0.81, indicating a high level of internal consistency [12].

This study utilized the GAD-7 scale to assess the likelihood of anxiety symptoms in the respondents. The scale consists of seven self-reported items rated on a four-point Likert scale from 0 (not at all) to 3 (nearly every day), with total scores ranging from 0 to 21 [13]. A score of 10 or greater indicates a probable generalized anxiety disorder. The GAD-7 has demonstrated strong internal consistency and test–retest reliability and good criterion, construct, factorial, and procedural validity [14]. The reliability coefficient, Cronbach’s α, for the overall GAD-7 scale is 0.90, which is greater than the recommended value of 0.80, suggesting excellent reliability [15]

The well-being of respondents was explored using the WHO-5 Wellbeing Index, which consists of five items rated on a six-point Likert scale [16,17]. The scores range from 0 to 25, with 0 indicating the lowest well-being and 25 indicating the highest well-being. For this study, a cut-off score below 13 was used to indicate poor mental well-being [16]. The BRS was utilized to evaluate the level of participants’ resilience, specifically their ability to recover from stress. Scores from 1.00 to 2.99 denote low resilience, whereas scores from 3.00 to 5.00 indicate normal-to-high resilience [18]. The BRS has demonstrated good internal consistency, with Cronbach’s alphas ranging from 0.80 to 0.90 and test–retest reliability coefficients over two weeks ranging from 0.61 to 0.69 [18,19].

The RAS is a 20-item measure developed based on a process model of recovery, which evaluates various aspects of recovery with a special focus on hope and self-determination. It has a satisfactory internal reliability (Cronbach α range = 0.73–0.91) [20]. The ACE Questionnaire is a 10-item measure used to measure childhood trauma. The questionnaire assesses 10 types of childhood trauma measured in the ACE Study. It has good internal consistency (Cronbach’s alpha = 0.76) [21].

The BSCS is a self-reporting scale used to assess the intensity, frequency, and length of time spent craving in the past 24 h, using a five-point Likert scale ranging from 0 to 4 with a mean score of 0 indicating no cravings in the past 24 h and a mean score of 4 indicating a high tendency for drug cravings. It has a high degree of internal consistency (Cronbach’s alpha 0.85–0.94) [22]

The C-SSRS consists of six questions assessing suicidal thoughts and behaviors. Respondents answer “Yes” or “No” to questions about suicidal ideation, plans, or attempts. Each question evaluates a unique aspect of suicide risk, with scoring classified as Low, Moderate, or High based on positive responses. If “Yes” is answered for Question 2, respondents proceed to Questions 3–5; otherwise, they skip to Question 6. A “Yes” to any question suggests potential referral to a mental health professional, while a “Yes” to Questions 4, 5, or 6 indicates high risk. The C-SSRS is widely validated and supported by organizations like the Substance Abuse and Mental Health Services Administration (SAMHSA), Centers for Disease Control and Prevention (CDC), Food and Drug Administration (FDA), National Institutes of Health (NIH), and WHO [23].

### 2.5. Data Analysis

Descriptive statistics were used to summarize the sociodemographic and clinical characteristics of participants from both the RASP and CMHP. Frequencies and percentages were calculated for categorical variables, and the mean and standard deviations were reported for continuous variables. Chi-square tests were conducted to assess significant differences between the two groups for categorical data, with Cramer’s V and Phi as measures of effect size. Independent *t*-tests were used for continuous variables following a normality test to ensure the data met the necessary assumptions. A significance level of *p* < 0.05 was set for all statistical analyses. Where necessary, Bonferroni correction was applied to adjust for multiple comparisons and reduce the risk of Type I errors.

### 2.6. Ethical Considerations

This study was conducted following the Declaration of Helsinki, and ethical approval was obtained from the NSH Research Ethics Board (REB File #: 1028254) and the Text4Support study (REB File #: 1028174).

## 3. Results

A comparison of the sociodemographic and clinical characteristics of individuals accessing services through the RASP and CMHP reveals several key differences, as displayed in Table 1.

The sample consisted of 1392 participants, with 36.9% identifying as male and 63.1% as female.

The findings displayed in Table 1 suggest that there was no significant difference in sex distribution between the RASP and CMHP groups. However, a significant difference was observed in gender distribution, with a higher proportion of individuals identifying as non-binary or “Other” in the RASP group. A significant association was found between education level and group membership. Individuals in the CMHP group were more likely to have only a high school education and less likely to hold a college or university degree compared to those in the RASP group. Employment status also differed significantly between groups. Unemployment was more common in the CMHP group, whereas employment and retirement were more frequent in the RASP group.

Relationship status varied significantly between the two groups. The CMHP group had a higher proportion of single participants, while the RASP group had a greater proportion of partnered or married individuals. The ethnicity distribution was significantly different between groups. The RASP group had a higher proportion of Caucasian participants, whereas the CMHP group had more individuals identifying as Indigenous or from other ethnic backgrounds. Income levels were significantly associated with group membership. The CMHP group had a larger proportion of participants with lower incomes, while the RASP group had a greater proportion of those in higher income brackets. Housing status also showed a significant difference between groups. Participants in the CMHP group were more likely to rent or live with family or friends, while homeownership was more common in the RASP group.

A significant difference was observed in psychiatric diagnoses. Depression was more prevalent in the RASP group, whereas the CMHP group had higher rates of substance use disorder, alcohol use disorder, psychosis, and other mental health conditions. There was no significant difference between groups regarding thoughts of dying, as both groups reported similar proportions. However, significant differences were observed in suicidal thoughts, with the CMHP group reporting a higher proportion of individuals experiencing them.

Notwithstanding the above findings, a Bonferroni correction was applied to adjust the significance threshold and account for multiple comparisons, thereby reducing the likelihood of false positives (Type I errors). With the adjusted *p*-value set at 0.005, formal educational level, employment status, relationship status, ethnicity, income range, housing, and diagnosis remained statistically significant. However, sex, gender, and pronoun were no longer significant, as their *p*-values exceeded the adjusted threshold (*p*-value = 0.005).

This suggests that the observed differences in sex, gender, and pronoun usage between the two groups may be due to random chance, whereas the other variables continue to show strong evidence of an association with program type (RASP and CMHP).

As displayed in Table 2, a significant association was found regarding thoughts about methods of dying. While most participants in both groups did not report such thoughts, the CMHP group had a higher proportion of individuals who did. A significant difference was noted in the intention to act on suicidal thoughts. Almost all participants in the RASP group reported no intention to act, while a smaller majority in the CMHP group expressed the same.

Finally, a significant difference was observed between groups in terms of taking steps to plan a suicide. While most participants had not taken any steps, a greater proportion of the CMHP group had started working on a plan compared to those in the RASP group.

Inferring from Table 3, the independent sample *t*-tests revealed significant differences in several clinical characteristics between the two groups. The RASP group was significantly older on average compared to the CMHP group. Regarding adverse childhood experiences, the CMHP group reported significantly higher ACE scores than the RASP group. Similarly, resilience scores were significantly lower in the RASP group compared to the CMHP group.

In terms of mental health symptoms, RASP participants had significantly higher anxiety (GAD-7) and depression (PHQ-9) scores at program entry than CMHP participants. Self-perceived recovery, as measured by the RAS, was significantly lower in the RASP group compared to the CMHP group. Likewise, well-being scores (WHO-5) were significantly higher in the CMHP group than in the RASP group. Substance use patterns also showed significant differences between the groups. The CMHP group had significantly higher drug intensity, frequency, and length of use scores compared to the RASP group.

Overall, these findings suggest that participants in the RASP group presented with greater mental health symptom severity and lower well-being and resilience, while the CMHP group exhibited higher levels of substance use and greater exposure to adverse childhood experiences.

However, all the clinical variables remained statistically significant even after conducting a Bonferroni correction with the adjusted *p*-value set at 0.005. This indicates that the age differences, mental health scores, resilience, substance use measures, and well-being between RASP and CMHP groups, as evident in the *t*-test analysis, are not due to chance.

The high effect sizes (Cohen’s d) as recorded in Table 3 are an indication of the substantial differences between the groups for some of the clinical variables.

## 4. Discussion

The rationale for comparing the RASP and the CMHP is very essential given that, while these programs serve distinct purposes, the RASP focuses on rapid stabilization and early intervention and the CMHP provides long-term, comprehensive mental health support. They are both integral components of Nova Scotia’s mental health service continuum.

The comparative analysis of sociodemographic and clinical characteristics between individuals accessing mental health services through the RASP and CMHP highlights significant differences across various dimensions, including demographic, mental health, resilience, substance use, and overall well-being profiles. These findings highlight distinct participant profiles and emphasize the significance of tailored approaches in mental health programming to address the unique demographic, social, and clinical needs of each group effectively.

### 4.1. Gender and Education

While there were no significant differences in sex, the participants who accessed mental health care through the RASP included a higher proportion of individuals who identified as non-binary or “Other.” This finding aligns with data indicating that gender-diverse individuals often face unique mental health issues and are more likely to seek supportive and inclusive services [24,25]. Regarding the educational status of participants, the CMHP group had a greater proportion of participants with only a high school education, while the participants who accessed mental health through the RASP mostly had higher education levels. A major reason for the RASP group having a greater educational score compared to the CMHP may be that individuals with higher education and socioeconomic status may be better positioned to self-advocate, leading to a higher likelihood of referral to the RASP. Furthermore, this discrepancy may reflect differences in socioeconomic status or access to resources, which has implications for mental health service design. Lower educational status is frequently associated with socioeconomic disadvantages and can impact access to and engagement in mental health services [26].

### 4.2. Employment and Income

Employment status and income also varied significantly between the groups, with a greater unemployment rate and lower income levels observed in the CMHP group. The high rate of unemployment and low income levels in this group likely result from both clinical and socioeconomic factors. Severe mental health conditions such as substance use disorders and psychosis, which were more prevalent in the CHMP group, may have disrupted the consistency of employment and hence limited the job-seeking ability of the affected individuals. Additionally, lower educational levels, as seen in this group, inhibit securing high-paying jobs. These challenges collectively may have contributed to the higher unemployment and lower income levels observed among CMHP participants. This finding aligns with data that state that persons with lower income and higher unemployment rates are more prone to experience financial and environmental difficulties, with a ripple effect in heightened mental health problems [27]. Furthermore, the employment differences suggest that the RASP group may have greater financial stability, enabling them to engage with services aimed at stabilization. On the other hand, CMHP participants may face more extensive social and economic challenges, highlighting a need for programs addressing economic barriers alongside mental health care [10].

### 4.3. Housing and Ethnicity

The higher proportion of homeownership in the RASP group compared to the CMHP group, where renting and living with family or friends were more common, further emphasizes socioeconomic differences. Stable housing is a protective factor against mental health problems, while unstable housing situations, such as living with family due to financial difficulties, are often associated with poor mental health outcomes [28]. Additionally, the RASP group had a greater proportion of Caucasian participants, whereas the CMHP group showed greater ethnic diversity, including a higher representation of Indigenous participants. This diversity in CMHP highlights the significance of culturally responsive and trauma-focused care within community-based mental health programs, particularly for marginalized populations who face historical and systemic mental health disparities [29].

### 4.4. Diagnoses and Mental Health Symptom Profile

There was a significant difference in primary diagnoses presented by the participants, with the RASP group showing a higher rate of depression. In contrast, the CMHP group presented higher rates of substance use disorders, psychosis, and alcohol use disorders. This distribution aligns with the intended design of each program, with RASP addressing acute stabilization needs for severe mental health symptoms. Another possible explanation for this trend is that referring clinicians may perceive the RASP as more suitable for patients with mood and anxiety disorders, while those with psychotic or substance use disorders are directed toward the CMHP, which offers longer-term support. Additionally, individuals experiencing acute psychosis or significant substance use challenges may require specialized interventions that are not currently integrated into the RASP, potentially limiting its accessibility to these populations.

Higher rates of suicidal thoughts, intentions to act, and working on plans in the CMHP group may imply that CMHP participants face persistent and severe mental health issues, potentially associated with substance use and other chronic disorders, which need to be explored in future studies. These findings are consistent with an earlier study that suggests that substance use and psychosis increase suicidal thoughts and behaviors [30].

Individuals who accessed mental healthcare through the RASP were significantly older compared to the CMHP group. Given that age is often associated with different support systems, and treatment needs in mental healthcare settings, the observed age difference may suggest that the rapid access program attracts mental health seekers who are further along their life course and may have faced more prolonged or recurrent psychological problems requiring immediate and intensive support. The differences in age between the two groups could account for the variations in the mental health outcomes and the levels of resilience, as older individuals often display a unique pattern in mental health and coping compared to younger individuals [31,32].

The higher ACE scores, as seen in the CMHP group, suggest a greater prevalence of early-life trauma, which is known to have enduring effects on individuals’ mental health and increases the risk of developing psychiatric disorders [33,34,35]. Additionally, in contrast to mental health symptom severity, the CMHP group displayed higher scores on the BSCS and longer histories of substance use. This may indicate that the CMHP attracts people whose primary concerns involve substance use disorders and who benefit from the integrated substance use treatment services available within the CMHP. Substance use is a significant factor in community mental health settings, and the higher substance-related scores underscore the importance of tailored substance-focused resources within the CMHP [36,37].

The findings revealed higher GAD-7 and PHQ-9 scores in the RASP group, suggesting elevated levels of anxiety and depression compared to the CMHP group. Given the severity of these symptoms, the RASP’s higher scores imply that it serves a population with more acute mental health needs that require rapid intervention and stabilization, as opposed to the potentially less severe cases seen in the CMHP. Anxiety and depression scores have been associated with the need for specialized, targeted interventions [38,39], which support the rationale for the establishment of the RASP as an acute response framework.

Finally, this study evaluated the mean difference in the resilience and well-being of participants within the two groups under study. Compared to the CMHP, individuals who accessed mental health support through the RASP had lower scores on the BRS. This low score may reflect diminished resilience, possibly because of more chronic or treatment-resistant mental health conditions. Previous studies have established that resilience is a protective factor against anxiety and depressive symptoms, particularly in populations with exposure to adversity or ongoing mental health challenges [40,41]. On the other hand, the lower scores on the WHO-5 Well-being Index in the RASP group further highlight this group’s reduced sense of well-being. Such a combination of low resilience and well-being may indicate the need for more intensive support and interventions focused on building coping mechanisms, which aligns with the services the RASP aims to provide.

### 4.5. Clinical Significance of This Study

This direct comparison was valuable because it allowed us to identify the distinct sociodemographic and clinical characteristics of individuals accessing each program. Additionally, understanding the differences in patient profiles may offer insights into referral patterns, service utilization, and potential gaps in care, ultimately contributing to improved coordination and efficiency across the mental health care system.

The unique demographic and clinical characteristics of the RASP and CMHP participants highlight the importance of program-specific interventions that address their different needs. CMHP participants, facing greater economic challenges, housing instability, and higher rates of substance use and psychosis, could benefit from a more integrated approach that includes mental health, housing, employment, and social services support. Trauma-informed care may also be beneficial, given the higher ACE scores within this group, potentially improving resilience and engagement. Conversely, RASP participants, who are more socioeconomically stable, would benefit from a focus on crisis stabilization and recovery practices intended to manage severe depression and anxiety. By identifying these unique sociodemographic and clinical distinctions, mental healthcare providers, stakeholders, and mental health program developers can optimize engagement and outcomes, ultimately supporting a more effective, equitable mental healthcare system. Future studies should aim to incorporate qualitative designs to explore these lived experiences and consider the impact of tailored interventions—such as resilience-building strategies for RASP participants and trauma-informed care for CMHP participants, especially those with higher adverse childhood experience scores. Addressing these different needs may improve clinical outcomes, resilience, and patient satisfaction for both groups.

### 4.6. Limitations and Future Directions

While this study presents valuable insights, several limitations must be acknowledged. First, the cross-sectional design adopted restricts causal inferences, and longitudinal research would be more valuable in evaluating how sociodemographic and clinical factors influence engagement and outcomes over time across the participants in both groups (the RASP and CMHP). Again, the reliance on self-reported data may have introduced the potential for recall bias and social desirability bias, which may have affected the accuracy of responses. Additionally, the quantitative focus of this study, while informative, may lack qualitative insights into the individual participants’ experiences, which may offer a deeper understanding of their specific needs and enhance interventions. This study further acknowledges the limitations of Phi (φ) and Cramér’s V, particularly their reliance on sample size, which may have inflated the effect sizes. Future studies should explore whether targeted outreach, expanded referral criteria, or integrated services within the RASP could improve access for individuals with conditions such as psychosis and substance use disorders, thereby enhancing the program’s ability to serve a broader range of patients requiring rapid stabilization.

## 5. Conclusions

The differences observed between the RASP and CMHP participants reflect unique characteristics and risk factors that mental health programs should address to enhance service efficacy. From the study, the RASP primarily serves a relatively older, socioeconomically stable population requiring acute stabilization for conditions marked by a greater prevalence of anxiety, depression, and lower resilience. RASP participants may require cognitive behavioral therapy and resilience-building interventions. On the other hand, the CMHP seems to support a more diverse, economically disadvantaged, and clinically complex population with a higher prevalence of early trauma and substance use, indicating a need for trauma-informed and substance-focused care. Identifying and addressing these differences through targeted, program-specific interventions may improve engagement and outcomes across both programs, advancing mental health and social equity and ultimately reducing wait times while increasing access to mental healthcare in Nova Scotia.

## Figures and Tables

**Table 1:** Baseline distribution of sociodemographic characteristics between the two programs (RASP and CMHP).

Variable		RASP N (%)	CMHP N (%)	Total N (%)	X^2^_(df)_	*p*-Value	Phi/Cramer’s V
Sex	Male	347 (36.3)	167 (38.3)	514 (36.9)	0.52 (1)	0.47	−0.02
	Female	609 (63.7)	269 (61.7)	878 (63.1)			
Gender	Male	340 (35.7)	167 (38.3)	507 (36.5)	8.36 (2)	0.02	0.08
	Female	598 (62.8)	253 (58.0)	851 (61.3)			
	Other	14 (1.5)	16 (3.7)	30 (2.2)			
Pronoun	He/his	338 (36.0)	165 (37.8)	503 (36.6)	9.16 (2)	0.10	0.08
	She/hers	586 (62.4)	253 (58.0)	839 (61.0)			
	Other	15 (1.6)	18 (4.1)	33 (2.4)			
Formal educational level	Elementary	14 (1.5)	6 (1.4)	20 (1.4)	30.18 (4)	<0.001	0.15
	High School	257 (27.0) * −5.4	181 (41.6) * 5.4	438 (31.6)			
	College/University	582 (61.1) * 4.7	207 (47.6) * −4.7	789 (56.9)			
	Post-secondary (trade school)	79 (8.3)	32 (7.4)	111 (8.0)			
	Other	20 (2.1)	9 (2.1)	29 (2.1)			
Employment status	Student	76 (8.0)	40 (9.2)	116 (8.4)	32.44 (4)	<0.001	0.15
	Employed	526 (55.3) * 2.6	209 (47.9) * −2.6	735 (53.0)			
	Unemployed	157 (16.5) * −4.0	112 (25.7) * 4.0	269 (19.4)			
	Retired	97 (10.2) * 3.8	18 (4.1) * −3.8	115 (8.3)			
	Other	95 (10.0)	57 (13.1)	152 (11.0)			
Relationship	Single	367 (38.6) * −6.7	252 (57.9) * 6.7	619 (44.6)	52.37 (4)	<0.001	0.19
	Partnered/Married	472 (49.6) * 5.7	144 (33.1) * −5.7	616 (44.4)			
	Divorced/Separated	85 (8.9) * 2.0	25 (5.7) * −2.0	110 (7.9)			
	Widower	20 (2.1)	5 (1.1)	25 (1.8)			
	Other	8 (0.8) * −1.9	9 (2.1) * 1.9	17 (1.2)			
Ethnicity	Indigenous	34 (3.6)	24 (5.5)	58 (4.2)	34.09 (7)	<0.001	0.16
	Black (African descent)	29 (3.1)	20 (4.6)	49 (3.6)			
	East Asian	12 (1.3)	4 (0.9)	16 (1.2)			
	Latino	3 (0.3)	3 (0.7)	6 (0.4)			
	Middle East	10 (1.1)	4 (0.9)	14 (1.0)			
	South Asian	5 (0.5)	4 (0,9)	9 (0.7)			
	Caucasian	833 (89.2) * 4.1	354 (81.2) * −4.1	1187 (86.6)			
	Other	8 (0.9) * −5.1	23 (5.3) * 5.1	31 (2.3)			
Income Range	No income	102 (11.4)	53 (12.2)	155 (11.7)	64.19 (5)	<0.001	0.22
	<29,590	271 (30.3) * −6.1	207 (47.5) * 6.1	478 (35.9)			
	29,592 to 59,180	272 (30.4)	129 (29.6)	401 (30.2)			
	59,181 to 93,000	171 (19.1) * 5.0	37 (8.5) * −5.0	208 (15.6)			
	93,001 to 150,000	58 (6.5) * 4.1	6 (1.4) * −4.1	64 (4.8)			
	More than 150,000	20 (2.2)	4 (0.9)	24 (1.8)			
Housing	Own home	348 (36.5) * 7.1	77 (17.7) * −7.1	425 (40.6)	60.97 (5)	<0.001	0.21
	Rented Accommodation	388 (40.7) * −3.8	224 (51.5) * 3.8	612 (44.1)			
	Live with family/friends	192 (20.1) * −2.3	112 (25.7) * 2.3	304 (21.9)			
	Couch surfing	7 (0.7) * −2.2	9 (2.1) * 2.2	16 (1.2)			
	Shelter/street	1 (0.1) * −3.1	6 (1.4) * 3.1	7 (0.5)			
	Other	17 (1.8)	7 (1.6)	24 (1.7)			
Diagnosis	Depression	385 (48.2) * 8.0	84 (19.3) * −8.0	469 (34.3)	170.87 (7)	<0.001	0.35
	Anxiety	237 (25.4) * 2.1	88 (20.2) * −2.1	325 (23.7)			
	Bipolar disorder	111 (11.9)	42 (9.7)	153 (11.2)			
	Substance use disorder	18 (1.9) * −5.3	34 (7.8) * 5.3	52 (38.8)			
	Alcohol use disorder	5 (0.5) * −5.2	20 (4.6) * 5.2	25 (1.8)			
	Trauma-related disorder	90 (9.6)	45 (10.3)	135 (9.9)			
	Psychosis	23 (2.5) * −6.4	46 (10.6) * 6.4	69 (5.0)			
	Other	65 (7.0) * −6.0	76 (17.5) * 6.0	141 (10.3)			

NB: * = Z-SCORE, Phi (φ) was used for 2 × 2 tables, while Cramér’s V was applied for larger contingency tables.

**Table 2:** Differences in suicidal ideation between RASP and CMHP.

Variable CSSRS		RASP N (%)	CMHP N (%)	Total N (%)	*p*-Value	X^2^_(df)_	Phi/Cramer’s V
Have you wished you were dead?	No	536 (56.2)	236 (54.1)	772 (55.6)	0.49	0.54 (1)	0.02
	Yes	417 (43.8)	200 (45.9)	617 (44.4)			
Have you had the thought of killing yourself?	No	827 (86.8)	282 (64.7)	1109 (79.8)	<0.001	90.78 (1)	0.26
	Yes	126 (13.2)	154 (35.3)	280 (20.2)			
Have you been thinking about how you will die?	No	879 (92.2)	340 (78.0)	1219 (87.8)	<0.001	56.58 (1)	0.20
	Yes	74 (0.8)	96 (22.0)	170 (12.2)			
Have you had these thoughts and had some intention of acting on them?	No	947 (99.4)	355 (81.4)	1302 (93.7)	<0.001	164.14 (1)	0.34
	Yes	6 (0.6)	81 (18.6)	87 (6.3)			
Have you started to work out or worked out the details of how to kill yourself?	No	944 (99.1)	384 (88.1)	1328 (95.6)	<0.001	85.93 (1)	0.25
	Yes	9 (0.9)	52 (11.9)	61 (4.4)			

NB: Phi (φ) was used for 2 × 2 tables, while Cramér’s V was applied for larger contingency tables.

**Table 3:** Independent sample *t*-test.

Variables	Program	N	Mean Score	S.D.	Mean Differences	95% CI	T Value (df)	*p* Value	Effect Size (Cohen d)
AGE	RASP	951	40.10	14.91	5.58	4.00–7.17	6.91 (1385)	<0.001	14.0
	CMHP	436	34.52	11.61					
ACE	RASP	930	3.16	2.58	−0.76	−1.08–−0.43	−4.58 (1276)	<0.001	2.63
	CMHP	348	3.92	2.78					
BRS	RASP	926	2.47	0.76	−0.31	−0.40–−0.22	−6.85 (1347)	<0.001	0.76
	CMHP	423	2.77	0.78					
GAD-7	RASP	934	14.17	5.61	2.36	1.69–3.04	6.87 (1357)	<0.001	5.88
	CMHP	425	11.81	6.42					
PHQ-9	RASP	908	16.62	6.28	2.43	1.67–3.19	6.28 (1329)	<0.001	6.57
	CMHP	423	14.20	7.15					
RAS	RASP	912	74.93	14.03	−6.78	−8.48–−5.09	−7.84 (1336)	<0.001	14.75
	CMHP	426	81.71	16.18					
WHO−5	RASP	942	6.37	4.63	−3.38	−0.96–−2.90	−11.50 (1371)	<0.001	5.05
	CMHP	431	9.75	5.97					
BSCS_Drug Intensity	RASP	360	2.69	1.23	−0.40	−0.62–−0.16	−3.36 (530)	<0.001	1.26
	CMHP	172	3.09	1.33					
BSCS_Drug frequency	RASP	354	2.81	1.20	−0.37	−0.59–−0.15	−3.27 (524)	<0.001	1.22
	CMHP	172	3.18	1.25					
BSCS_Drug length time	RASP	353	2.41	1.18	−0.49	−0.72–−0.27	−4.30 (523)	<0.001	1.24
	CMHP	172	3.91	1.36					

## Data Availability

The datasets used and/or analyzed during the current study are available from the corresponding author upon reasonable request.
